# Establishing CRISPR/Cas13a immune system conferring RNA virus resistance in both dicot and monocot plants

**DOI:** 10.1111/pbi.13095

**Published:** 2019-03-14

**Authors:** Tong Zhang, Yaling Zhao, Jiajie Ye, Xue Cao, Chenhui Xu, Biao Chen, Hong An, Yuting Jiao, Fushun Zhang, Xin Yang, Guohui Zhou

**Affiliations:** ^1^ Guangdong Province Key Laboratory of Microbial Signals and Disease Control College of Agriculture South China Agricultural University Guangzhou Guangdong China; ^2^ Integrative Microbiology Research Centre South China Agricultural University Guangzhou Guangdong China; ^3^ Division of Biological Sciences University of Missouri Columbia MO USA

**Keywords:** CRISPR/Cas13a system, RNA virus, resistance, dicot plants, monocot plants

Dear Editor,

Besides its powerful capability for genome editing, the clustered, regularly interspaced short palindromic repeats (CRISPR)/CRISPR‐associated (Cas) (CRISPR/Cas) systems, has been exploited to combat virus infection in eukaryotic organisms (Zaidi *et al*., 2016). By harnessing CRISPR/Cas system, its compelling inhibiting activities against DNA viruses (Ali *et al*., 2015; Baltes *et al*., 2015; Ji *et al*., 2015) or RNA viruses (Aman *et al*., 2018; Zhang *et al*., 2018) were reported in many cases. Moreover, due to the facts that the eukaryotic viruses themselves do not equip the ability to counter this prokaryotic immune defense, by utilization of such strategy, we could establish effective control and eradiation strategy against the eukaryotic virus. For dicot plant, the systems were well‐established and reported. However, for monocot plants, encompassing many important grain crops, whose yield was significantly influenced by serious viral diseases, no effective and valid method has been reported by using CRISPR/Cas system to build safeguard for them against viruses.

RNA viruses cause serious losses in crops and significant damage to agricultural production, and there are two types of CRISPR/Cas effectors, Cas9 from *Francisella novicida* (FnCas9) and Cas13a from *Leptotrichia shahii* (LshCas13a) or *Leptotrichia wadei* (LwaCas13a), have been introduced to target RNA *in vivo* (Abudayyeh *et al*., 2016; Sampson *et al*., 2013). Previously we have successfully established FnCas9 immune system conferring RNA virus in tobacco and *Arabidopsis* (Zhang *et al*., 2018), in this study, we reprogrammed and expressed the LshCas13a system in plants, and employed several RNA viruses to test the antivirus effect of the CRISPR/Cas13a system from *L. shahii*. Our study demonstrates this system can target and degrade viral RNA genomes, and confer resistance to RNA viral diseases in monocot grain plants.

To establish invading RNA virus resistance in plants, we constructed pCambia1300‐derived vectors pCR11 and pCR12, which were used to express the CRISPR/Cas13a machinery from *L. shahii* driven by suitable promoters for dicot or monocot plants, respectively (Figure 1a).

**Figure 1:**
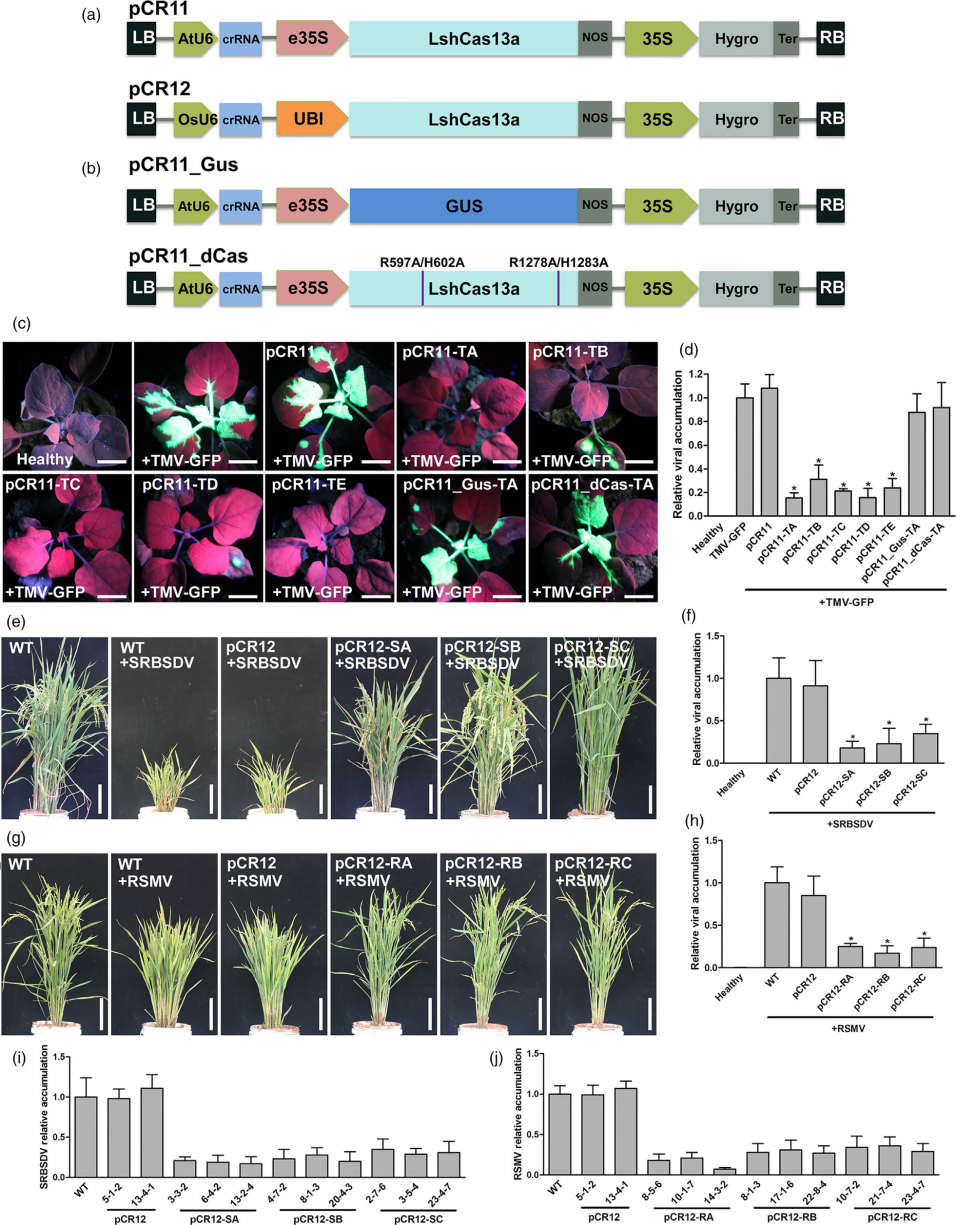
RNA virus resistance was established in dicot and monocot plants by using CRISPR/Cas13a. (a) pCR11 and pCR12 were used to express the LshCas13a and crRNA driven by suitable promoters for dicot or monocot plants, respectively. (b) Variants of pCR11 used in this study. pCR11_GUS was used to express the crRNA only, in which the LshCas13a which is substituted by a GUS gene. pCR11‐dCas was used to express the crRNA with a mutant LshCas13a which was cleavage deficient. (c) Transient expression of the *Leptotrichia shahii* CRISPR/Cas13a system in *Nicotiana benthamia*na conferred resistance to TMV. Tobacco plants were infiltrated with TMV‐GFP together with pCR11‐crRNAs or control vectors. At 7 dpi, plants were imaged under UV light for GFP of each inoculating combination. Scale bars = 2 cm. (d) TMV accumulation levels in *N. benthamiana* systemic leaves were assessed RT‐qPCR. (e) Transgenic rice plants established resistance to SRBSDV. The symptoms of T1 pCR12‐crRNAs or control vectors transgenic rice plants after SRBSDV inoculation. Scale bars = 10 cm. (f) SRBSDV accumulation in the transgenic plants was assessed by RT‐qPCR. (g) Transgenic rice plants established resistance to RSMV. The symptoms of T1 pCR12‐crRNAs or control vectors transgenic rice plants after RSMV inoculation. Scale bars = 10 cm. (h) RSMV accumulation in the transgenic plants was assessed by RT‐qPCR. Error bars represent SD. All the data were representative of three biological replicates.

*Tobacco mosaic virus*, which is a classic virus infecting dicot plants, was employed to evaluate the defense efficiency of the CRISPR/Cas13a system. Five crRNAs targeting TMV genome were synthesized and inserted into pCR11 to create corresponding pCR11‐crRNA vectors. A recombinant *Tobacco mosaic virus* agro‐infectious clone which is expressing GFP (TMV‐GFP) and the pCR11‐crRNA vector were simultaneously injected into 25‐day‐old *Nicotiana benthamiana* leaves. At 1 week post‐inoculation, bright green fluorescence was observed in control plants, which were inoculated by TMV‐GFP only, or TMV‐GFP plus pCR11 (Figure 1c). In pCR11‐TA, pCR11‐TB, pCR11‐TC, pCR11‐TD and pCR11‐TE inoculated tobacco plants, the green fluorescence was obviously weaker compared with control (Figure 1c), which reflects that the TMV infection was significantly attenuated by the CRISPR/Cas13a system. Quantification of the TMV titre by RT‐qPCR further confirmed that the TMV‐GFP levels in CRISPR targeted plants were significantly decreased (Figure 1d).

To exclude the possibility that the crRNA bind to the viral genome and inhibit the infection without the help of LshCas13a, a GUS gene was substituted for the LshCas13a to produce pCR11_Gus (Figure 1b). The pCR11_Gus‐TA, which is targeting the TMA TA site, lost the ability to suppress the TMV‐GFP infection as pCR11‐TA (Figure 1c and d). Then, alanine point mutations in the two higher eukaryotes and prokaryotes nucleotide‐binding (HEPN) RNases domains of LshCas13a (R597A, H602A, R1278A and H1283A; Abudayyeh *et al*., 2016), were generated to test whether the endonucleolytic activity was involved in the repression of virus infection (Figure 1b). The pCR11_dCas‐TA abrogated the repression of TMV‐GFP infection compared with pCR11‐TA (Figure 1c and d). These results indicate that the cleavage sites of LshCas13a were essential for inhibiting virus infection in our system.

Plant viruses not only harm dicot plants, but also monocot plants, such as rice, which suffered serious yield losses by many viruses. For example, *Southern rice black‐streaked dwarf virus* (SRBSDV) causes a striking disease on rice in several East Asian countries (Zhou *et al*., 2013). Here we synthesized three crRNAs targeting the double strand RNA genome of SRBSDV and inserted into pCR12. The resulting vectors, pCR12‐SA, pCR12‐SB, pCR12‐SC and along with the control vector pCR12 were transformed into rice plants mediated by agrobacterium. T1 transgenic lines for each construct, along with control wild‐type rice plants, were selected and infected with SRBSDV by its viruliferous vector feeding. Forty days later, typical symptoms were observed in the control plants, including significant dwarfing and failure to head (Figure 1e). In the transgenic lines, most plants showed mild symptoms, and the pCR12‐SB lines in particular had no obvious symptoms (Figure 1e). Quantification of virus accumulation by RT‐qPCR showed that SRBSDV infection was indeed inhibited in these transgenic plants (Figure 1f). Rice Stripe Mosaic Virus (RSMV) is a novel cytorhabdovirus and became a new threat to rice production in south China (Yang *et al*., 2017). We also generate transgenic rice plants harbouring the CRISPR/Cas13a system targeting the single strand RNA genome of RSMV. Virus attacking experiment showed that the control plants had typical symptoms, including slight dwarfing, with leaves showing yellow stripes and excessive tillering, while the transgenic plants with crRNA targeting RSMV (pCR12‐RA, pCR12‐RB and pCR12‐RC) had very mild symptoms (Figure 1g) and less viral RNA accumulation(Figure 1h). To test the inheritability of the resistance, we harvest the T3 homozygous lines to attack by SRBSDV or RSMV. Inspiringly, all the T3 transgenic plants we tested showed stable resistance to SRBSDV (Figure 1i) or RSMV (Figure 1j). Our results showed that overexpressing of crRNA–LshCas13a specifically targeting the viral genome was an effective way to generate stable RNA virus resistance in monocot plants.

In the past decades, RNA interference‐mediated resistance has been used to confer immunity against viruses in plants. However, through long‐term co‐evolution, eukaryotic viruses have developed methods of antagonize RNAi, which limited the applications in agriculture. In recent years, the CRISPR/Cas9 machinery has been exploited to combat eukaryotic viruses in dicot plants (Ali *et al*., 2015; Aman *et al*., 2018; Baltes *et al*., 2015; Ji *et al*., 2015; Zhang *et al*., 2018). To our knowledge, this is the first report of a method targeting the viral RNA to control viral diseases in monocot plants. In addition, we have used two distinct type of CRISPR/Cas system, Cas9 from *F. novicida* (Zhang *et al*., 2018) and Cas13a from *L. shahii* (this study), both of them showed high efficiency in generating RNA virus‐resistant plants. The difference is that the former depends on FnCas9 binding viral RNA, while the latter requires LshCas13a having RNases activity to cleave viral RNA. These findings provide us with more options for developing antiviral strategies, and combination of multiple strategies may provide reference to generate viral‐immune crops in the future.

Our findings demonstrate that the *L. shahii* CRISPR/Cas13a system we established in this study could enable the plant acquire potent defense against viral infection in both dicot and monocot plants, which imply that the method has the potential to develop into a universal applicable system in various kinds of crop species.

## Competing interests

T.Z. and G.Z. have filed a patent application in China (priority filing with serial number 201811493466.4).

## Author contributions

T.Z. and G.Z. designed the experiments; T.Z., Y.Z., J.Y., X.C., C.X., B.C., H.A., Y.J. and F.Z. performed the experiments; T.Z., Y.Z., H.A. and G.Z. analysed the results; T.Z. and G.Z. wrote the manuscript. All authors read and approved the final manuscript.

## References

[pbi13095-bib-0001] Abudayyeh, O.O. , Gootenberg, J.S. , Konermann, S. , Joung, J. , Slaymaker, I.M. , Cox, D.B.T. , Shmakov, S. *et al*. (2016) C2c2 is a single‐component programmable RNA‐guided RNA‐targeting CRISPR effector. Science, 353, aaf5573. 27256883 10.1126/science.aaf5573 PMC5127784

[pbi13095-bib-0002] Ali, Z. , Abulfaraj, A. , Idris, A. , Ali, S. , Tashkandi, M. and Mahfouz, M.M. (2015) CRISPR/Cas9‐mediated viral interference in plants. Genome Biol. 16, 1–11. 26556628 10.1186/s13059-015-0799-6 PMC4641396

[pbi13095-bib-0003] Aman, R. , Ali, Z. , Butt, H. , Mahas, A. , Aljedaani, F. , Khan, M.Z. , Ding, S. *et al*. (2018) RNA virus interference via CRISPR/Cas13a system in plants. Genome Biol. 19, 1. 29301551 10.1186/s13059-017-1381-1 PMC5755456

[pbi13095-bib-0004] Baltes, N.J. , Hummel, A.W. , Konecna, E. , Cegan, R. , Bruns, A.N. , Bisaro, D.M. and Voytas, D.F. (2015) Conferring resistance to geminiviruses with the CRISPR–Cas prokaryotic immune system. Nature Plants, 1, 15145. 34824864 10.1038/nplants.2015.145 PMC8612103

[pbi13095-bib-0005] Ji, X. , Zhang, H. , Zhang, Y. , Wang, Y. and Gao, C. (2015) Establishing a CRISPR–Cas‐like immune system conferring DNA virus resistance in plants. Nature Plants, 1, 15144. 27251395 10.1038/nplants.2015.144

[pbi13095-bib-0006] Sampson, T.R. , Saroj, S.D. , Llewellyn, A.C. , Tzeng, Y.‐L. and Weiss, D.S. (2013) A CRISPR/Cas system mediates bacterial innate immune evasion and virulence. Nature, 497, 254–257. 23584588 10.1038/nature12048 PMC3651764

[pbi13095-bib-0007] Yang, X. , Huang, J. , Liu, C. , Chen, B. , Zhang, T. and Zhou, G. (2017) Rice stripe mosaic virus, a novel cytorhabdovirus infecting rice via leafhopper transmission. Front. Microbiol. 7, 2140. 10.3389/fmicb.2016.02140 28101087 PMC5210121

[pbi13095-bib-0008] Zaidi, S.S.‐e.‐A. , Tashkandi, M. , Mansoor, S. and Mahfouz, M.M. (2016) Engineering plant immunity: using CRISPR/Cas9 to generate virus resistance. Front. Plant Sci. 7, 1673. 27877187 10.3389/fpls.2016.01673 PMC5099147

[pbi13095-bib-0009] Zhang, T. , Zheng, Q. , Yi, X. , An, H. , Zhao, Y. , Ma, S. and Zhou, G. (2018) Establishing RNA virus resistance in plants by harnessing CRISPR immune system. Plant Biotechnol. J. 16, 1415–1423. 29327438 10.1111/pbi.12881 PMC6041442

[pbi13095-bib-0010] Zhou, G. , Xu, D. , Xu, D. and Zhang, M. (2013) Southern rice black‐streaked dwarf virus: a white‐backed planthopper‐transmitted fijivirus threatening rice production in Asia. Front. Microbiol. 4, 270. 24058362 10.3389/fmicb.2013.00270 PMC3766826

